# A Global Estimate of the Number of Coral Reef Fishers

**DOI:** 10.1371/journal.pone.0065397

**Published:** 2013-06-19

**Authors:** Louise S. L. Teh, Lydia C. L. Teh, U. Rashid Sumaila

**Affiliations:** Fisheries Centre, University of British Columbia, Vancouver, British Columbia, Canada; Swansea University, United Kingdom

## Abstract

Overfishing threatens coral reefs worldwide, yet there is no reliable estimate on the number of reef fishers globally. We address this data gap by quantifying the number of reef fishers on a global scale, using two approaches - the first estimates reef fishers as a proportion of the total number of marine fishers in a country, based on the ratio of reef-related to total marine fish landed values. The second estimates reef fishers as a function of coral reef area, rural coastal population, and fishing pressure. In total, we find that there are 6 million reef fishers in 99 reef countries and territories worldwide, of which at least 25% are reef gleaners. Our estimates are an improvement over most existing fisher population statistics, which tend to omit accounting for gleaners and reef fishers. Our results suggest that slightly over a quarter of the world’s small-scale fishers fish on coral reefs, and half of all coral reef fishers are in Southeast Asia. Coral reefs evidently support the socio-economic well-being of numerous coastal communities. By quantifying the number of people who are employed as reef fishers, we provide decision-makers with an important input into planning for sustainable coral reef fisheries at the appropriate scale.

## Introduction

How many fishers do coral reefs support? This question is important to fisheries managers and coastal planners throughout coral reef regions. Coral reefs support the nutritional and economic needs of fishers in many of the world’s poorest and most vulnerable communities [1], [2]; yet, they are threatened by human activities and climate events [3]–[6]. Decisions have to be made to balance protecting coral reefs and allowing people to use them for social and economic purposes. Fishing is perhaps the most direct form of human dependence, as well as stressor, on coral reefs. Socio-economic drivers such as market access, poverty, lack of appropriate institutions, and population growth influence overfishing of reef fisheries [7]–[9]. Management measures to address overfishing require a baseline on which to measure outcomes – the most basic is the number of participants in a reef fishery. However, there is a general lack of knowledge about the number of people who fish on coral reefs. This paper’s objective is to fill the identified information gap by providing a global estimate of the number of coral reef fishers.

We use the term coral reef fisheries to encompass fishing on coral reefs as well as associated habitats such as seagrass meadows, which often make up an important component of the reef fishing area [10]. Reef fisheries typically tend to be small-scale, artisanal and/or subsistence in nature, and operate in rural or remote places away from regulated landing sites. Poverty or social status may limit fishers’ ability to obtain fishing licenses. Thus, coral reef fisheries are generally not monitored regularly and a large portion of coral reef fishers are unaccounted for by national governing authorities [11]. At the same time, even small-scale reef fisheries that take place close to urban areas are often overlooked by governments, which tend to focus resources on regulating and managing larger-scale export oriented fisheries [12]. For these reasons, the number of coral reef fishers worldwide is largely unknown, but ignoring this sector can hamper management efforts to meet societal needs under changing environmental, social and economic conditions.

Ensuring food security and adapting to climate change are among the primary challenges facing coastal nations today [13]. Coral reef fisheries help to maintain food security and social stability, as a portion of reef catches are generally kept to feed fishers’ families, or to share with friends and neighbours [14], [15]. Information on the number of coral reef fishers can be used to assess fishing pressure on near shore habitat, including coral reefs, and to project if there is adequate capacity to supply fish protein to meet local and national demands.

A number of studies have shown that fisher densities (number of fishers per area) affect the ecological state of coral reef fisheries. For instance, [16] found that trophic levels and fish size decreased with higher fisher densities at fishing grounds in Papua New Guinea. Similarly, gear type used and ecological state varied along a gradient of fisher densities in Kenya [9], while [17] found that predatory fish were lower at islands with highest fishing intensity. These studies highlight the importance of knowing coral reef fisher populations for social and ecological management. However, this information is not readily available neither on a global, nor in many cases, a national or sub-national level.

We compile a database of the number of coral reef fishers in all countries where coral reefs occur. Our estimate encompasses the number of coral reef fishers in the primary sector, i.e., those who catch fish, either full-time or part-time, for both artisanal and subsistence purposes. It also includes women and children who typically glean in shallow inshore areas for edible coral reef organisms. This global study provides an estimate of reef fisher numbers at the country level. We recognise that data at this scale may not be suitable for the development of community level interventions, which require finer scale studies that estimate undocumented fisher populations [e.g., 18]. Nevertheless, the estimates presented here provide a starting point for identifying potential countries where extra attention to finer scale studies is warranted.

### Context

The socio-economic importance of coral reefs cannot be overstated. Annual net economic benefits derived from healthy coral reef ecosystems are estimated at around USD 30 billion a year, with reef fisheries contributing USD 5.7 billion of this [19]. Over 400 million people in the poorest developing countries worldwide live within 100 km of coral reefs, and of these, the majority live in rural settings where they tend to be most directly dependent on reef resources to support their livelihoods and food security [20].

Reef fisheries are intensely targeted not only for a local source of protein, but also for export oriented trades such as the live reef food fish, aquarium, and curio trades [21], [22]. Consequently, there is high fishing pressure on coral reefs, with more than 55% of the world’s coral reefs considered threatened, and almost 30% highly threatened by overfishing and/or destructive fishing [5]. On top of this, coral reefs are further stressed by climate change events as well as land based development and pollution [11], [23]. The combined impact of multiple stressors may compromise the ability of coral reefs to continue supporting the food and income needs of coastal communities in the future [6], [11], [24].

**Figure 1:**
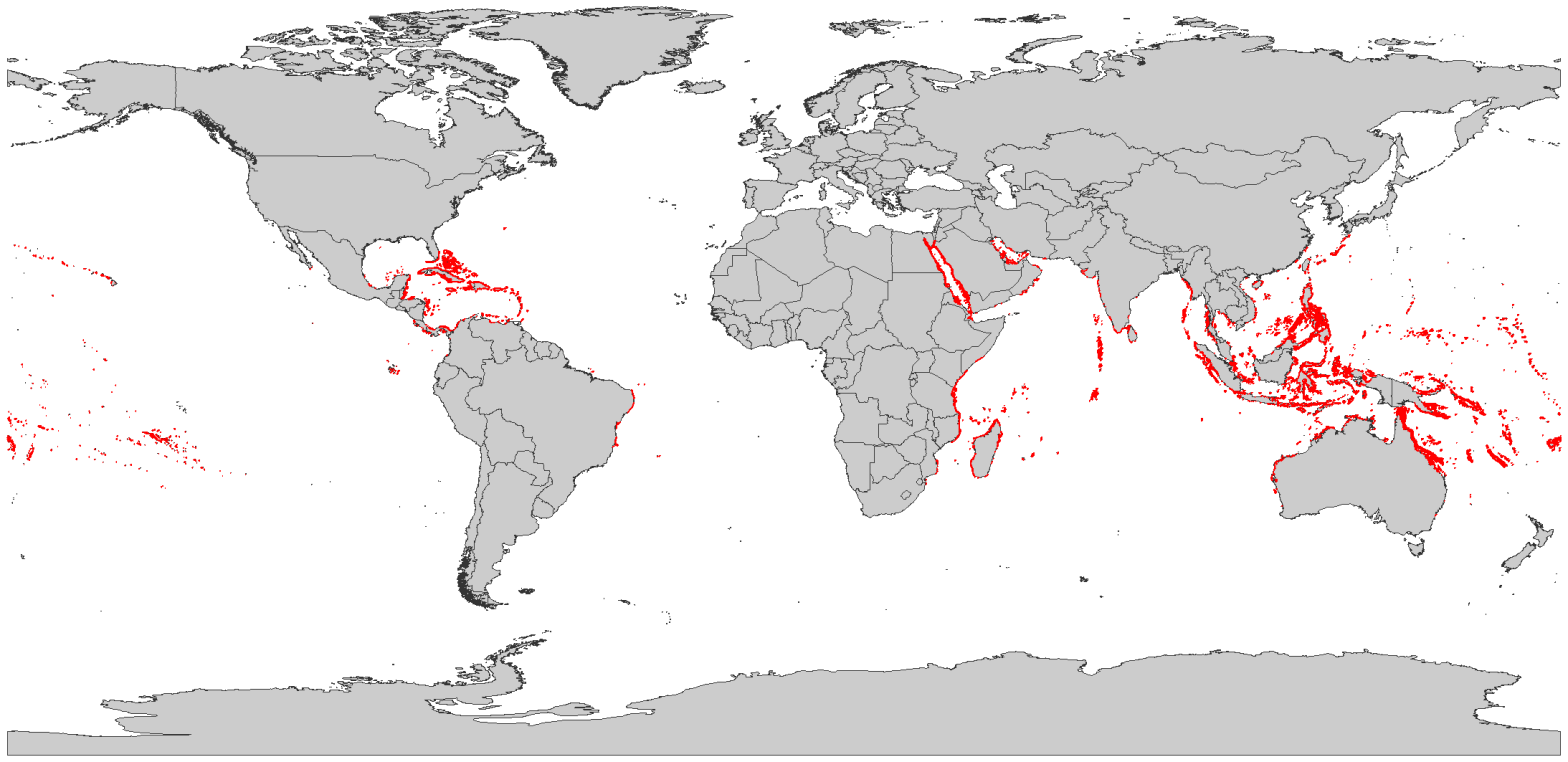
Map of global coral reef distribution. **Coral reefs are outlined in red.** Source: UNEP-WCMC (http://datda.unep-wcmc.org).

Around 100 countries and territories in the world have coral reefs (Fig. 1), with Indonesia, Australia, and the Philippines possessing the highest proportions of total coral reef area, at 18%, 17%, and 9%, respectively [25]. Over 80% of the world’s coral reefs are concentrated in Asia and Oceania. Of the coral reef countries and territories with United Nations Human Development Index (UN HDI) ratings, roughly 36% are ranked “High” or “Very High”, 50% are ranked “Medium” and 13% are ranked “Low” [26].

Coral reef fisheries are multispecies and multigear in nature. Fishing is usually undertaken by small-scale fishers who use unmotorised or low powered boats and manually operated gears such as hand lines, nets, traps, and spears. In many regions, destructive fishing methods such as the use of fish bombs and sodium cyanide are also widespread on coral reefs [27]. Reef fisheries have been overexploited globally [28], with declines in catch rates and changes in species composition reported throughout reef countries [9], [16], [29], [30]. Despite the heavy fishing pressure on coral reefs, there has, to date, been no comprehensive global estimate of the number of reef fishers.

In 2006, approximately 3 million people worldwide, almost entirely from countries or territories with coral reefs, collected sea cucumbers [31]. Further, it was estimated that there were 6.2 million small-scale fishers from 16 countries in 2000 (including Indonesia and the Philippines), and that 15–35% of small-scale catches came from coral reefs [2]. If we assume that catch composition is representative of fisher composition, then between 930,000 and 2.2 million fishers in the 16 studied countries may be considered as coral reef fishers.

## Methods

### Coverage of Coral Reef Countries and Territories

We estimated the number of fishers in all coral reef countries, identified according to the UN Atlas of Coral Reefs [25], inclusive of overseas territories with coral reef area. In total, our study covered 100 reef countries and territories.

### Estimating the Number of Reef Fishers

We used two approaches for estimating the number of reef fishers, which we chose based on the availability of published literature, and on the following premise:

1. Reef fishers constitute a fraction of a reef country’s total marine fishers. We assumed that this fraction is represented by the ratio of reef fish to total marine fish landed value of a country or territory;
2. The number of reef fishers is a function of reef area, coastal population, and fishing pressure.

#### Approach 1: Proportion of reef-related to total marine fish landed values

We assumed that reef fishers are a subset of all marine fishers (inclusive of small and large scale) in each reef country or territory. An estimate of the number of fishers worldwide is reported in [32]. As there is no existing metric indicating what proportion of fishers in coral reef countries are reef fishers, we adopted the % of reef fish landed value/total marine fish landed value as a rough approximation of reef fishers out of total fishers. We chose to use landed value because it better reflects fishers’ incentive to fish - for a specified volume of fish, more fishers would target the higher value fish. We recognise that reef fisheries landings and therefore landed values are not well monitored and likely underreported [2], [33], thereby making our estimate of reef fishers to be on the low side.

We determined the percentage of total landed value attributable to reef fish species for each reef country in 2005 [34]. Landed value data were obtained from the *Sea Around Us* Project (SAUP) catch database (www.seaaroundus.org), except for Florida, which was not reported separately in the database. We identified 200 reef fish species and taxon groups in the SAUP catch database (Table S1 in File S1). Reef associated pelagics such as scombrids and carangids normally form part of reef fish catches [35]. However, we chose not to include reef associated pelagics as they are also targeted and caught in greater quantities by larger scale, non-reef operations. Therefore, in order not to overestimate the proportion of reef fishers, we included inshore reef species only. The reef/total marine landed value % (Table S2 in File S1) was applied to the number of total marine fishers in each reef country or territory to obtain the number of reef fishers:
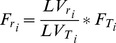
(1.1)


Where 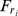 is the number of reef fishers in country *i*


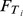
 is the total number of marine fishers in country *i* from [32]


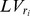
 is the landed value of reef fish catch in country *i*


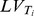
 is the landed value of total marine fish catch in country *i*

According to the SAUP catch database, the following reef countries had no coral reef species listed in their 2005 catches and hence, no landed values: Bangladesh, Brunei, Cambodia, Cayman Islands, Comoros, Djibouti, Dominica, Dominican Republic, Federated States of Micronesia, Marshall Islands, Mayotte, Mozambique, Myanmar, Nauru, New Caledonia, Niue, Solomon Islands, Sudan, Tokelau, and Vietnam. We first searched the literature to ascertain whether reef fisheries were present in these countries and territories. Marine catches in Cambodia, Brunei, and Singapore are insignificant [36]. Due to the well developed economy of Singapore and its relatively low coral reef area, we assumed that it had no reef fisheries. Aside from Singapore, reef fisheries were present in the other countries and territories listed above. For these cases, we applied the regional average of reef/total marine landed value % obtained from the SAUP database as the default reef landed value % (Table 1).

**Table 1:** Average reef/total marine landed value % by region.

Region	Regional average reef catch %
Middle East	43
East Pacific/Atlantic	40
Indian Ocean	18
Western Pacific	14
Southeast Asia	11

#### Approach 2: Fisher density

We calculated the number of reef fishers by multiplying fisher density (no. of fishers per km^2^ of fishing ground) by the reef area of each country or territory. Fisher density in each country was defined by two variables: i) rural coastal population size; and ii) fishing pressure:
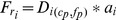
(1.2)



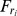
 is the number of coral reef fishers in country *i*;


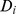
 is fisher density of country *i* stratified by rural coastal population (*c_p_*) and fishing pressure (*f_p_*);


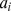
 is coral reef area in country *i.*

Reef area (*a_i_*) was based on estimates reported in the UN Coral Reef Atlas [25]. Fisher density (*D_i_*) for each reef country or territory was defined by rural coastal population and fishing pressure exerted on their respective coral reefs. Previous studies have shown a correlation between human population levels and fishing effort in coral reef areas [7], [37], [38]. Estimating a mathematical relationship between the increase in rural coastal population and fishing pressure was not possible with existing data. We thus assumed a positive linear relationship between the two variables, and found it reasonable to equate high rural coastal population in coral reef countries with having high fisher population, hence high fisher density. High fishing pressure was taken as an indication of high fishing effort, which we equated with high fisher density [9]. As reef fishers worldwide tend to use manually operated gears [35], we found it reasonable to assume that high fishing effort arose from more people fishing rather than due to the use of more efficient fishing gear.

### Rural Coastal Population Data

Rural coastal population data were extracted from the Socioeconomic Data and Applications Center’s (SEDAC) Global Rural-Urban Mapping Project [39]. The dataset provides estimates of rural population in the low elevation coastal zone in 2000, where low elevation is defined as land 10 meters or less in elevation. Rural low elevation coastal zone populations in 2000 were then projected to 2010 by tracking average population growth rates from 2000 to 2010 [40]. Next, we categorised each nation’s rural coastal population as being high, medium, or low (Table 2). We considered all nations with rural coastal populations less than the 50^th^ percentile (45,000) to be ‘low’; population levels between the 50–75^th^ percentile were considered to be ‘medium’, while those above the 75^th^ percentile were ‘high’.

**Table 2:** Summary of rural coastal population level, and level of fishing pressure on coral reefs for coral reef countries and territories worldwide.

Country	Rural coastal population level	Fishing pressure level	Source
American Samoa	Low	High	[28]
Anguilla	Low	Low	[28]
Antigua and Barbuda	Low	High	[28]
Aruba	Low	Low	[28]
Australia	Medium	Low	[27]
Bahamas	Medium	Medium	[28]
Bahrain	Medium	High	[28]
Bangladesh	High	High	[28]
Barbados	Low	High	[28]
Belize	Low	Low	[42]
Bermuda	Low	High	[28]
Brazil	High	High	[27]
British Virgin Islands	Low	High	[28]
Brunei Darussalam	Low	Low	[27]
Cambodia	High	High	[27]
Cayman Islands	Low	High	[27]
China	High	High	[27]
Colombia	High	Medium	[42]
Dominica	Low	High	[28]
Comoros	Medium	High	[28]
Cook Islands	Low	Low	[28]
Costa Rica	Medium	High	[5]
Cuba	High	Medium	[28]
Djibouti	Low	Low	[27]
Dominican Republic	Medium	High	[42]
Ecuador	Low	High	[66]
Egypt	High	High	[5]
Eritrea	Low	Low	[27]
Federated States of Micronesia	Low	High	[28]
Fiji	Medium	High	[28]
Florida & US Gulf of Mexico	High	High	[27]
French Polynesia	Low	Low	[28]
Grenada	Low	High	[28]
Guadeloupe	Low	High	[28]
Guam	Low	High	[28]
Haiti	High	High	[42]
Hawaii	High	High	[27]
Honduras	Medium	High	[43]
India	High	High	[27]
Indonesia	High	High	[27]
Iran	High	Low	[27]
Israel	Low	Low	[27]
Jamaica	Medium	High	[28]
Japan	High	Medium	[27]
Jordan	Low	Low	[27]
Kenya	Medium	High	[27]
Kiribati	Medium	Low	[28]
Kuwait	Medium	Medium	[27]
Madagascar	High	High	[28]
Malaysia	High	Medium	[27]
Maldives	Medium	Low	[28]
Marshall Islands	Low	Low	[28]
Martinique	Low	High	[28]
Mauritius	Low	High	[28]
Mayotte	Low	Low	[28]
Mexico	High	High	[5]
Mozambique	High	High	[27]
Myanmar	High	Medium	[27]
Nauru	Low	Medium	[28]
Netherland Antilles	Low	Low	[28]
New Caledonia	Low	Low	[28]
Nicaragua	Medium	Low	[5]
Niue	Low	Low	[28]
Northern Mariana Islands	Low	Medium	[27]
Oman	Low	High	[27]
Palau	Low	Medium	[28]
Panama	Medium	Medium	[27]
Papua New Guinea	Medium	Low	[28]
Philippines	High	High	[28]
Puerto Rico	Medium	High	[27]
Qatar	Low	Low	[27]
Reunion	Medium	High	[28]
St. Kitts and Nevis	Low	High	[42]
St. Lucia	Low	High	[42]
St. Vincent and the Grenadines	Low	High	[42]
Samoa	Low	High	[28]
Saudi Arabia	Medium	Low	[27]
Seychelles	Low	High	[28]
Solomon Islands	Medium	Medium	[28]
Somalia	Medium	Low	[27]
Sri Lanka	High	High	[28]
Sudan	Medium	Medium	[27]
Taiwan	High	High	[27]
Thailand	High	Medium	[27]
Tokelau	Low	Low	[28]
Tonga	Low	High	[28]
Trinidad and Tobago	Low	High	[28]
Turks and Caicos Islands	Low	Medium	[28]
Tuvalu	Low	Low	[28]
United Arab Emirates	Low	Medium	[27]
Tanzania	High	High	[27]
United States Virgin Islands	Low	High	[28]
Vanuatu	Low	Low	[28]
Venezuela	Medium	High	[27]
Vietnam	High	High	[27]
Wallis and Futuna	Low	Low	[28]
Yemen	Medium	High	[27]

### Fishing Pressure Assessment

The level of fishing pressure was determined based on mainly qualitative assessments derived from 3 primary publications: Reefs at Risk Series [5], [41], Status of Coral Reefs in the World Series [27], [42], and Newton et al. (2007) [28] . We used global assessments rather than going by a case by case basis in order to minimise the variability in assessment methods across nations and territories. The general analytical approach used in these publications is briefly summarised below:

- Reefs at Risk Series: These studies used GIS (Geographic Information System) to map where reefs were at risk from degradation due to local threats, including overfishing, and global threats, such as climate change. The threat to coral reefs from overfishing was evaluated based on coastal population data, extent of fishing areas, occurrences of destructive fishing, and presence of marine protected areas. The level of threat was also informed by input from coral reef scientists and experts. The risk levels were low, medium, high, and very high;
- Status of Coral Reefs in the World: These reports relied on the expert opinions of local reef scientists and managers from around the world to determine the status of each nation’s coral reefs, and the extent to which reefs were threatened by human activities such as overfishing;
- Newton et al. (2007): Unlike the above 2 studies which assessed the level of threat to coral reefs from overfishing, this study evaluated the status of 49 reef fisheries worldwide. Each reef fishery was categorised as being underexploited, overexploited, or fully exploited, based on estimates of how much national reef fish landings exceeded maximum sustainable yield from coral reefs.

Each country was categorised as having high fishing pressure if its reef fishery was assessed to be overexploited in [28], or if the country’s reefs were determined to be at high or very high risk or threat from overfishing by [27] or [5] and [41] (Table 2). Medium fishing pressure was assigned to those nations with fully exploited reef fisheries according to [28], or where coral reefs were at medium levels of risk or threat from overfishing. Lastly, nations whose fisheries were determined to be underexploited by [28], or were at low risk or threat from overfishing in [27] or [5] and [41], were considered to have low fishing pressure (Table 2).

### Fisher Density Data

Fisher density levels (fishers⋅km^−2^ of fishing ground) corresponding to inshore small scale fisheries were assigned to nations that were now segregated by rural coastal population and fishing pressure. For instance, American Samoa was assigned a fisher density level of 8.6 fishers⋅km^−2^ (Table 3), as it had a low rural coastal population level and high fishing pressure (Table 2). Fisher density levels in high and medium coastal population countries were based on case studies of countries with corresponding levels of coastal populations, in this case, Philippines [43] and Kenya [9], respectively. The majority (74%) of island nations in the Western Pacific had low rural coastal population levels; therefore, we based fisher density levels for low coastal population countries on case studies of the Pacific islands. Recent studies on reef resource use were available for 6 low population Pacific islands - the Marshall Islands, French Polynesia, New Caledonia, Wallis and Futuna, Tuvalu, and the Cook Islands [44]–[49].

**Table 3:** Fisher density (No. of fishers per km^2^ fishing ground) for reef fisheries in countries/territories with high, medium, and low coastal populations, and under high, medium, or low fishing pressure.

	Coastal population		
Fishing Pressure	High	Medium	Low
High^1^	36	21.8	8.6
Medium^2^	19	13.5	5.9
Low^3^	2	5.2	3.5

1 Source: [44].

2 Source: [9].

3 Source: [45]–[50].

In the Kenya case study, mean fishing densities for three fisheries management areas ranged from 5 to 22 fishers⋅km^−2^. We used the maximum, minimum, and midpoint (13.5) of this range to represent high, low, and medium fisher densities (Table 3). Fishing at these sites mainly took place from shore to the outer reef and fringing reef lagoon. Fishing effort in Kenya was typically concentrated in shallow, hard bottom back reef locations that were 0.5–3 m deep at low tide [50]. The Philippines case study provided fisher density levels in 25 coastal communities which ranged widely from 1 to 131 fishers⋅km^−2^ of fishing ground. These fisheries took place in shallow coastal areas within 15 km from shore. To avoid potential outliers, we excluded the two extreme values, resulting in a narrower range of 2 to 36 fishers⋅km^−2^. As with Kenya, we used the maximum, minimum, and midpoint (19) to represent high, low, and medium fisher densities. We applied the same procedure of eliminating extreme endpoint values in the 6 Pacific island case studies. We then took the averaged maximum, minimum, and midpoint fisher density values across all 6 cases to represent high, low, and medium fisher densities (8.6, 5.9, and 3.5 fishers⋅km^−2^ of fishing ground) in low rural coastal population countries (Table 3). Fishing in these Pacific islands took place on sheltered coastal reefs and lagoons, outer reefs, and passages, which ranged in depths from 0–18 m.

### Number of Gleaners

We assumed that fisher density levels did not account for those who glean on intertidal reef flats or in shallow water habitats such as seagrass meadows, because the gears and targeted species of fishers described in the source material were not consistent with gleaning. To account for gleaners, we identified countries where a substantial portion of the population engages in gleaning, and then doubled the number of coral reef fishers estimated by the fisher density level approach only. We did not have to account for gleaning in the first approach (reef fishers as a proportion of total marine fishers) because gleaners were already included in the base estimate from [32].

Countries or territories were considered to have substantial gleaning if they were mainly subsistence based or if fishing was part of their cultural identity. Based on these criteria, all island nations in the Western Pacific were determined to have substantial gleaning. We chose a 1∶1 gleaner to coral reef fisher ratio because gleaning is usually done by women and children for subsistence purposes. Therefore, we conservatively assumed that in places where gleaning is widespread, each fishing household had at least one other member besides the fisher who gleaned.

We further identified countries where some gleaning took place, but not at as high a rate as in the Pacific islands. These countries were extracted from [31], who assessed the status of sea cucumber fisheries worldwide as being subsistence, small-scale, industrial, or illegal. We assumed that if gleaning for sea cucumbers existed, then gleaning for other invertebrates was also practised. Thus, all countries that were assessed as having subsistence, small-scale, or illegal sea cucumber fisheries by [31] were treated as having a gleaning sector. We omitted sea cucumber fisheries categorised as ‘industrial’ (e.g., China, Iceland, and Western Canada) because those lacked a traditional or subsistence aspect, therefore it was unlikely that community members besides professional fishers participated. Partial gleaning countries were then allocated a gleaner to fisher ratio based on their UN Human Development Index (HDI) rank, with the rationale that higher HDI ranked countries or territories (i.e., those with higher standard of living) would have less necessity to rely on gleaning, which is usually a subsistence or supplementary income activity. Due to the lack of global scale gleaning effort data, we arbitrarily defined gleaner to fisher proportions as follows: Very high HDI = 0%; High HDI = 10%; Medium HDI = 30%; Low HDI = 50%.

### Calculation and Verification of Number of Fishers

The final number of coral reef fishers in 99 countries (excluding Singapore) was then calculated as the average of approach 1 (proportion of total marine fishers by landed value) and 2 (fisher density), with the results from each approach defining the upper and lower range. To verify whether our estimate was within reasonable bounds, we conducted an *ad hoc* search of the literature for data or indicators relating to coral reef fisher numbers (e.g. fisher employment, number of small-scale fishers, proportion of fishing households) to determine whether our estimates were within similar orders of magnitude. More systematically, we calculated the proportion of coral reef fishers to rural coastal population in each country or territory. It was reported by [5] that the country with highest relative participation in reef fishing (New Caledonia) involved 40% of the population. Given that 43% of New Caledonia’s population is rural, and assuming that all reef fishing takes place in rural areas, the reef fishing participation rate is approximately equivalent to 70% of the rural population. We applied this rate as the upper limit of reef fishing participation in rural coastal populations, although we recognise that participation may actually be higher given that our upper limit was derived from total, rather than coastal population.

We used 70% as a cut-off for all countries and territories except for small island nations in the Western Pacific, where nearly all households in coastal villages are involved in fishing [51]. An exception was Fiji, for which we used the number of reef fishers provided by [52] because the author provided a detailed national estimate of subsistence and artisanal fishers, including gleaners. For all other cases that exceeded the 70% limit, reef fisher numbers were assigned based on reported figures from the literature (Table S3 in File S1). It should be noted that it was not possible to ascertain whether reef gleaners were included in these reported figures.

### Sensitivity Analysis

Approach 1: The average percentage of reef related to total marine fish landed value across all 99 countries and territories was 0.28. We applied this to the total number of fishers of each country or territory to derive the number of coral reef fishers. We also tested a maximum and minimum of 50% and 10%, given that the highest and lowest regional average reef/total marine landed value percentages were 43% and 11%, respectively.

Approach 2: We multiplied the coral reef area for each country or territory by the overall average fisher density across all countries and territories (14.7 fishers⋅km^−2^). We also tested how results changed if all reef fisheries were considered to be i) underexploited (fisher density of 5.2 fishers⋅km^−2^); and ii) overexploited (fisher density of 21.5 fishers⋅km^−2^), using the fisher densities corresponding to medium population levels.

Gleaners: We tested two scenarios. The high scenario involved doubling the current gleaning participation rates in all countries and territories where gleaning is present, while the low scenario halved the participation rate.

## Results and Discussion

The total estimated number of fishers worldwide was 6.1 million, with a possible range from 5.2 million (fisher density approach) to 6.8 million (reef to total marine landed value proportional approach). The estimated 6 million reef fishers represent about 2% of the 275 million people who live within 30 km of reefs around the world [5]. Southeast Asia had the highest number of reef fishers (3.35 million), followed by the Indian Ocean (1.5 million). The Middle East had the lowest number of reef fishers (344,459) (Table 4). Indonesia was the country with the highest number of reef fishers (1.7 million), and was the only country where reef fishers exceeded a million. India and the Philippines had the next two highest populations of reef fishers, with 959,000, and 912,000, respectively (Table S3 in File S1). When viewed as a proportion of reef fishers to rural coastal population, however, Western Pacific island nations had the highest percentage of reef fishers (68%), while Southeast Asia, at 5%, had the lowest proportion of reef fishers (Table 4). We found that subsistence gleaning took place in about 70% of reef countries or territories, and reef gleaners made up at least 25% of total reef fishers. Southeast Asia accounted for 60% of gleaners, while both the Western Pacific and Indian Ocean regions accounted for 16% of reef gleaners each.

**Table 4:** Estimated number of reef fishers in coral reef regions worldwide.

Region	Number of reef fishers (million)	Reef fishers as % of rural coastal population
Southeast Asia	3.35	5
Indian Ocean	1.50	13
E. Pacific/Atlantic	0.50	18
Western Pacific	0.45	68
Middle East	0.34	24
**TOTAL**	**6.14**	

A recent study estimated 3 million sea cucumber fishers worldwide [31]. As the majority of these invertebrate fisheries take place on or near coral reefs, sea cucumber fishers can be viewed as a subset of reef fishers. An earlier study by [2] suggested between 930,000 to 2 million reef fishers in 16 countries, including Indonesia and the Philippines, which are two of the most populous reef countries. Both these studies provide an indication that our global estimate of 6 million reef fishers is within a reasonable range. Moreover, there are an estimated 2.25 million fishers in the Coral Triangle [53], which coincides fairly well with our estimate of 2.8 million fishers in the 6 Coral Triangle countries of Malaysia, Indonesia, Philippines, Solomon Islands, Papua New Guinea, and Timor-Leste.

In total, fisher estimates for 11 countries exceeded the 70% rural population limit, and these were replaced with estimates from alternate sources (Table S3 in File S1). For corroboration, we obtained alternate reef fisher estimates for 12 additional countries or territories (Table S4 in File S1). Our approach generated a higher estimate for 7 of 12 (58%) of these countries. Overall, the sum of fishers from these 23 countries was 37% higher using our approach compared to the sum obtained from the independent studies. However, it is noted that the reef fisher estimates from alternate sources were mainly from the early 2000s; thus, it is likely fisher numbers could have increased in the past decade.

In the case of Timor-Leste, we used an alternate source even though the estimated number of reef fishers did not exceed 70%. This is because fishing in Timor-Leste is limited to inshore low-technology activities. Therefore, it was reasonable to assume that the number of fishers reported in [54] related to reef fishers.

For 2 cases (Guam and Papua New Guinea), only the fisher density approach was applied. For Guam, the standardised approach generated an estimate that exceeded the 70% cut-off. As we were unable to find an alternate source for Guam, we used the estimate generated by the fisher density method only, which did not exceed the 70% cut-off. The initial number of fishers estimated for Papua New Guinea (average of approach 1 and 2) did not exceed 70%.However, we chose to use the estimate provided by the fisher density method instead (based on 22 fishers per km^2^), although it exceeded the 70% cut-off. This is due to existing literature that reported about 116,000 people in Papua New Guinea’s artisanal fisheries [55], which exploit reef finfish, coastal pelagics, and sedentary reef species. The reported figure was a magnitude higher than our initial estimate, but close to that of the fisher density method, thus we selected the more representative estimate.

The 6 million reef fishers estimated here make up around 28% of the 22 million small-scale fishers globally [32]. Small-scale fishers already form one of the most marginalised segments of society [56], [57], and that more than one quarter of them fish primarily on coral reef ecosystems that are highly threatened by environmental change is of special concern. Particular attention should be focused on coral reef and reef fisheries management in Indonesia, India, and the Philippines, as 58% of the world’s reef fishers are concentrated in these 3 developing countries. This reinforces concerns about the future sustainability of reef fisheries in these and other densely populated reef countries [28], where poverty and other socio-economic pressures associated with developing countries intensify the prevalence of Malthusian overfishing [58], [59]. At the same time, high fisheries dependence on developing country coral reefs indicates that coral reef conservation has to explicitly consider the societal and economic cost of displacing reef fishing effort, and to consider livelihood based approaches that create incentives for communities to engage in conservation [60].

Although our estimate is coarse, it presents an improvement over existing sources that rely primarily on licensed fishers to estimate fisher population. Many reef fishers are not captured in official statistics since they live in isolated or rural communities [11]. Further, in regions such as Oceania, women and children gleaners who collect substantial amounts of coral reef food organisms are rarely considered to be ‘fishers’ [61], even though subsistence fishing contributes up to 70% of overall coastal fisheries production in the Pacific islands [52].

We recognise that more detailed data on reef fisher numbers are available from various socio-economic surveys conducted in coral reef communities. For instance, recent studies have compiled comprehensive socio-economic surveys in fishing communities in the Indian Ocean [62], Indo-Pacific [63], and Western Pacific islands [64]. However, these surveys tend to be done at the village level, and fisher numbers are not normally scaled up to a national level. Although they can be used to refine our numbers, our study is the first attempt to quantify the number of reef fishers worldwide using a consistent method. Further, it accounts for the active participation of women and children in reef gleaning, which is not normally accounted for in national statistics.

Overall, our estimate tends to be conservative. First, determining reef fishers based on reef catch landed value likely underestimates the proportion of reef fish relative to total marine landed value, due to widespread omission or underreporting of coral reef fisheries in official catch statistics [33]. Second, we accounted for reef gleaners by assuming a maximum of one gleaner per household. This is likely an underestimate since in countries where gleaning is widespread, it is typically undertaken by both the women and children in a household [1], [24]. Sensitivity analysis showed that the estimated number of reef fishers was not strongly influenced by changes to our assumptions about gleaners. Doubling the number of gleaning participants in all countries and territories increased the estimated number of reef fishers by 11%, whereas halving participation resulted in a decrease of 6%.

Instead, the estimated number of reef fishers was most sensitive to changes in the reef/total marine landed value percentage used in Approach 1. Using an overall average reef/total marine landed value proportion of 28% increased the estimated number of reef fishers worldwide by 43%. At the high end, applying a reef/total marine landed value percentage of 50% increased our estimate by 118% to about 14.5 million reef fishers, whereas it was reduced by 22% to approximately 5 million fishers when we applied a low end reef/total marine landed value percentage of 10%. In comparison, the fisher density approach produced a negligible increase if all reef fisheries were considered to be overexploited, and a decrease of 36% if all reef fisheries were assumed to be underexploited.

Improving on our estimate will necessitate a concerted effort by reef countries and territories to start collecting data on the number of people fishing on coral reefs. This is not an insurmountable task – the Global Coral Reef Monitoring Network already provides a comprehensive review on the status of coral reefs every 4 years [27]. It is plausible that this same network of scientists and institutions can also be used to start recording the number of reef fishers globally. The community-based monitoring conducted by the Global Socioeconomic Monitoring Initiative for Coastal Management (SocMon, www.socmon.org) also provides an opportunity for researchers to record the number of reef fishers in their surveys. Increasing focus on coral reef conservation can also be a means to record fisher numbers, as more non-governmental organisations initiate community conservation activities in coral reef dependent areas.

### Conclusion

The fact that small-scale fishers account for 90% of fishers worldwide [65] illustrates the relative importance of reef fisheries, which are small-scale by nature. Overfishing is the largest direct threat to coral reefs [5], but for national governments, a major impediment to reducing overfishing impacts is not knowing the magnitude of fishing pressure on coral reefs. Our study addresses this challenge by providing the first global estimate of the number of reef fishers, which, to our knowledge, is also the first to explicitly account for gleaners. At the national level, we provide reef and fisheries managers with a baseline estimate and demonstrate a methodology that can be used to overcome data poor situations to pursue more relevant and in-depth national estimates of reef fisher populations. This can then be applied to developing policies for regulating fishing effort on coral reefs, thereby helping to improve fishery management. By showing that over a quarter of the world’s small-scale fishers fish on coral reefs, our results place the socio-economic significance of reef fisheries into context at an international level. Overall, this study emphasizes that in order to sustain reef fisheries into the future, the cumulative effect fishing pressure is currently having on the world’s coral reefs has to be accounted for, even if it necessitates taking unconventional approaches such as those used here.

## Supporting Information

File S1 **Supporting tables.** Table S1. Reef taxa used to identify reef catch from the Sea Around Us catch database. Table S2. Reef/total landed value % for reef countries in 2005. Table S3. Estimated number of reef fishers for reef countries and territories worldwide in 2010. Table S4. Comparison of fisher estimates from current study and alternate independent sources.
(DOC)
