# Supplementary material for: A Global Estimate of the Number of Coral Reef Fishers
Source: PLoS One. 2013 Jun 19;8(6):e65397. doi: 10.1371/journal.pone.0065397 (PMC3686796; doi:10.1371/journal.pone.0065397)
Supplement: File S1 — Supporting tables. Table S1. Reef taxa used to identify reef catch from the Sea Around Us catch database. Table S2. Reef/total landed value % for reef countries in 2005. Table S3. Estimated number of reef fishers for reef countries and territories worldwide in 2010. Table S4. Comparison of fisher estimates from current study and alternate independent sources. (DOC) [file pone.0065397.s001.doc]

| Table S1. Reef taxa used to identify reef catch from the *Sea Around Us* catch database. | |
| --- | --- |
| **Taxon Name** | **Common Name** |
| Abudefduf luridus | Canary damsel |
| Acanthopagrus bifasciatus | Twobar seabream |
| Acanthopagrus schlegeli | Black porgy |
| Acanthuridae | Surgeonfishes and tangs and unicornfishes |
| Acanthurus sohal | Sohal surgeonfish |
| Aethaloperca rogaa | Redmouth grouper |
| Anadara | Anadara clams |
| Aphareus rutilans | Rusty jobfish |
| Apogonidae | Cardinalfishes |
| Arca | Ark clams |
| Archosargus probatocephalus | Sheepshead seabream |
| Atherinidae | Silversides |
| Balistes capriscus | Grey triggerfish |
| Balistidae | Triggerfishes |
| Bivalvia | Clams |
| Bolbometopon muricatum | Green humphead parrotfish |
| Bothidae | Lefteye flounders |
| Bothus pantherinus | Leopard flounder |
| Brachydeuterus auritus | Bigeye grunt |
| Caesionidae | Fusiliers |
| Calamus | Porgies |
| Cantherhines | Filefishes |
| Carcharhinidae | Requiem sharks |
| Carcharhinus brachyurus | Copper shark |
| Carcharhinus falciformis | Silky shark |
| Carcharhinus limbatus | Blacktip shark |
| Carcharhinus sorrah | Spottail shark |
| Caulolatilus princeps | Ocean whitefish |
| Centropomus | Snooks |
| Centropomus undecimalis | Common snook |
| Centropristis striata | Black seabass |
| Cephalopholis argus | Peacock hind |
| Cephalopholis fulva | Coney |
| Cephalopholis hemistiktos | Yellowfin hind |
| Cephalopholis miniata | Coral hind |
| Cheimerius nufar | Santer seabream |
| Chloroscombrus chrysurus | Atlantic bumper |
| Chromis chromis | Damselfish |
| Congridae | Conger and garden eels |
| Ctenolabrus rupestris | Goldsinny-wrasse |
| Dasyatidae | Stingrays |
| Dasyatis akajei | Red stingray |
| Diagramma pictum | Painted sweetlips |
| Echinoidea | Sea urchins |
| Elops saurus | Ladyfish |
| Ephippidae | Spadefishes and batfishes and scats |
| Epinephelus | Groupers |
| Epinephelus aeneus | White grouper |
| Epinephelus analogus | Spotted grouper |
| Epinephelus areolatus | Areolate grouper |
| Epinephelus chlorostigma | Brownspotted grouper |
| Epinephelus coioides | Orange-spotted grouper |
| Epinephelus fasciatus | Blacktip grouper |
| Epinephelus flavolimbatus | Yellowedge grouper |
| Epinephelus fuscoguttatus | Brown-marbled grouper |
| Epinephelus goreensis | Dungat grouper |
| Epinephelus guttatus | Red hind |
| Epinephelus marginatus | Dusky grouper |
| Epinephelus morio | Red grouper |
| Epinephelus morrhua | Comet grouper |
| Epinephelus multinotatus | White-blotched grouper |
| Epinephelus nigritus | Warsaw grouper |
| Epinephelus niveatus | Snowy grouper |
| Epinephelus polyphekadion | Camouflage grouper |
| Epinephelus striatus | Nassau grouper |
| Epinephelus summana | Summan grouper |
| Epinephelus tauvina | Greasy grouper |
| Galeocerdo cuvier | Tiger shark |
| Gnathanodon speciosus | Golden trevally |
| Gobiidae | Gobies |
| Gobius niger | Black goby |
| Gymnocranius | Large-eye breams |
| Gymnura altavela | Spiny butterfly ray |
| Haemulidae | Grunts |
| Holocentridae | Squirrelfishes and soldierfishes |
| Holothuroidea | Sea cucumbers |
| Kyphosus | Sea chubs |
| Labridae | Wrasses |
| Labrus bergylta | Ballan wrasse |
| Lateolabrax japonicus | Japanese seaperch |
| Lates calcarifer | Barramundi |
| Lethrinidae | Emperors or scavengers |
| Lethrinus atlanticus | Atlantic emperor |
| Lethrinus borbonicus | Snubnose emperor |
| Lethrinus harak | Thumbprint emperor |
| Lethrinus lentjan | Pink ear emperor |
| Lethrinus mahsena | Sky emperor |
| Lethrinus microdon | Smalltooth emperor |
| Lethrinus nebulosus | Spangled emperor |
| Lethrinus obsoletus | Orange-striped emperor |
| Lethrinus xanthochilus | Yellowlip emperor |
| Lutjanidae | Snappers |
| Lutjanus | Snappers |
| Lutjanus argentimaculatus | Mangrove red snapper |
| Lutjanus argentiventris | Yellow snapper |
| Lutjanus bohar | Two-spot red snapper |
| Lutjanus campechanus | Northern red snapper |
| Lutjanus gibbus | Humpback red snapper |
| Lutjanus johnii | Johns snapper |
| Lutjanus kasmira | Common bluestripe snapper |
| Lutjanus malabaricus | Malabar blood snapper |
| Lutjanus purpureus | Southern red snapper |
| Lutjanus quinquelineatus | Five-lined snapper |
| Lutjanus synagris | Lane snapper |
| Malacanthidae | Tilefishes |
| Monacanthidae | Filefishes |
| Monotaxis grandoculis | Humpnose big-eye bream |
| Mugil cephalus | Flathead mullet |
| Mugil liza | Liza |
| Mugilidae | Mullets |
| Mullidae | Goatfishes |
| Mulloidichthys flavolineatus | Yellowstripe goatfish |
| Mullus | Western goatfishes |
| Mullus argentinae | Argentine goatfish |
| Mustelus | Smooth-hounds |
| Mycteroperca | Cat groupers |
| Mycteroperca microlepis | Gag |
| Mycteroperca phenax | Scamp |
| Mycteroperca xenarcha | Broomtail grouper |
| Naso unicornis | Bluespine unicornfish |
| Nemipteridae | Threadfin breams and Whiptail breams |
| Nemipterus | Threadfin breams |
| Octopus vulgaris | Common octopus |
| Ocyurus chrysurus | Yellowtail snapper |
| Ostraciidae | Boxfishes (cowfish and trunkfish) |
| Panulirus | Tropical spiny lobsters |
| Panulirus argus | Caribbean spiny lobster |
| Panulirus cygnus | Australian spiny lobster |
| Panulirus gracilis | Blue spiny lobster |
| Panulirus homarus | Scalloped spiny lobster |
| Panulirus longipes | Longlegged spiny lobster |
| Paralichthys | American flounders |
| Pelates quadrilineatus | Fourlined terapon |
| Platax | Batfishes |
| Platycephalidae | Flatheads |
| Platycephalus indicus | Bartail flathead |
| Plectorhinchus gaterinus | Blackspotted rubberlips |
| Plectorhinchus pictus | Trout sweetlips |
| Plectorhinchus schotaf | Minstrel sweetlip |
| Plectropomus areolatus | Squaretail coralgrouper |
| Plectropomus leopardus | Leopard coralgrouper |
| Plectropomus pessuliferus | Roving coralgrouper |
| Plotosus | Eel catfishes |
| Pomacanthidae | Angelfishes |
| Pomacanthus maculosus | Yellowbar angelfish |
| Pomadasys argenteus | Silver grunt |
| Pomadasys kaakan | Javelin grunter |
| Portunus pelagicus | Blue swimming crab |
| Priacanthus | Bigeyes |
| Priacanthus macracanthus | Red bigeye |
| Pseudupeneus prayensis | West African goatfish |
| Rachycentron canadum | Cobia |
| Rhabdosargus haffara | Haffara seabream |
| Rhinobatidae | Guitarfishes |
| Rhomboplites aurorubens | Vermilion snapper |
| Rhynchobatus djiddensis | Giant guitarfish |
| Sargocentron spiniferum | Sabre squirrelfish |
| Saurida tumbil | Greater lizardfish |
| Saurida undosquamis | Brushtooth lizardfish |
| Scaridae | Parrotfishes |
| Scarus ghobban | Blue-barred parrotfish |
| Scarus persicus | Gulf parrotfish |
| Sciaenidae | Drums or croakers |
| Scolopsis | Monocle breams |
| Scolopsis taeniatus | Black-streaked monocle bream |
| Scorpaena | Scorpionfish |
| Scorpaenidae | Scorpionfishes or rockfishes |
| Scyliorhinus stellaris | Nursehound |
| Sea-urchins and other echinoderms | Sea urchins |
| Semicossyphus pulcher | California sheephead |
| Serranidae | Sea basses or groupers and fairy basslets |
| Serranus | Groupers |
| Sharks, rays, chimaeras | Sharks rays and skates etc |
| Siganus | Rabbitfishes |
| Sillaginidae | Smelt-whitings |
| Sillago sihama | Silver sillago |
| Sparidae | Porgies |
| Sphoeroides | Puffers |
| Sphoeroides maculatus | Northern puffer |
| Sphyrna lewini | Scalloped hammerhead |
| Sphyrna zygaena | Smooth hammerhead |
| Sphyrnidae | Hammerhead and bonnethead and scoophead shark |
| Starfish and other echinoderms | Starfishes |
| Stephanolepis cirrhifer | Thread-sail filefish |
| Strombus | Stromboid conchs |
| Symphodus melops | Corkwing wrasse |
| Synodontidae | Lizardfishes |
| Takifugu vermicularis | Pear puffer |
| Terapon | Trumpeters |
| Tetraodontidae | Puffers |
| Thalassoma pavo | Ornate wrasse |
| Triakidae | Houndsharks |
| Trichiuridae | Cutlassfishes |
| Upeneus | Tropical goatfishes |
| Valamugil seheli | Bluespot mullet |
| Variola louti | Yellow-edged lyretail |

| Table S2. Reef/total landed value % for reef countries in 2005. Landed value data are extracted from the *Sea Around Us* catch database (www.seaaroundus.org). | |
| --- | --- |
| **Country** | **Reef/Total Landed value** |
| American Samoa | 0.01 |
| Anguilla | 0.48 |
| Antigua and Barbuda | 0.95 |
| Aruba | 0.45 |
| Australia | 0.32 |
| Bahamas | 0.99 |
| Bahrain | 0.88 |
| Bangladesh* | 0.16 |
| Barbados | 0.01 |
| Belize | 0.80 |
| Bermuda | 0.46 |
| Brazil | 0.22 |
| British Virgin Islands | 0.41 |
| Brunei Darsm* | 0.38 |
| Cambodia* | 0.16 |
| Cayman Islands* | 0.44 |
| China | 0.04 |
| Colombia | 0.04 |
| Comoros* | 0.26 |
| Cook Islands | 0.03 |
| Costa Rica | 0.20 |
| Cuba | 0.59 |
| Djibouti* | 0.26 |
| Dominica* | 0.39 |
| Dominican Republic | 0.63 |
| Ecuador* | 0.16 |
| Egypt | 0.30 |
| Eritrea | 0.73 |
| Federated States of Micronesia | 0.01 |
| Fiji | 0.13 |
| French Polynesia | 0.04 |
| Grenada | 0.41 |
| Guadeloupe | 0.08 |
| Guam | 0.23 |
| Haiti | 0.27 |
| Honduras | 0.19 |
| India | 0.10 |
| Indonesia | 0.21 |
| Iran | 0.17 |
| Israel | 0.30 |
| Jamaica | 0.55 |
| Japan^1^ | 0.03 |
| Jordan | 0.18 |
| Kenya | 0.45 |
| Kiribati | 0.38 |
| Kuwait | 0.18 |
| Madagascar | 0.02 |
| Malaysia | 0.13 |
| Maldives | 0.01 |
| Marshall Islands* | 0.09 |
| Martinique | 0.42 |
| Mauritius | 0.72 |
| Mayotte | 0.00 |
| Mexico | 0.13 |
| Mozambique | 0.00 |
| Myanmar* | 0.16 |
| Nauru | 0.01 |
| Netherland Antilles | 0.01 |
| New Caledonia | 0.28 |
| Nicaragua | 0.53 |
| Niue* | 0.10 |
| Northern Mariana Islands* | 0.10 |
| Oman | 0.40 |
| Palau | 0.06 |
| Panama | 0.18 |
| Papua New Guinea | 0.01 |
| Philippines | 0.34 |
| Puerto Rico | 0.95 |
| Qatar | 0.72 |
| Reunion | 0.02 |
| St. Kitts and Nevis | 0.97 |
| St. Lucia | 0.09 |
| St. Vincent and the Grenadines | 0.004 |
| Samoa | 0.16 |
| Saudi Arabia | 0.62 |
| Seychelles | 0.04 |
| Solomon Islands* | 0.09 |
| Somalia | 0.06 |
| Sri Lanka | 0.05 |
| Sudan | 0.004 |
| Taiwan | 0.06 |
| Tanzania | 0.53 |
| Thailand | 0.18 |
| Timor-Leste | 0.02 |
| Tokelau* | 0.10 |
| Tonga | 0.09 |
| Trinidad and Tobago | 0.09 |
| Turks and Caicos Islands | 1.00 |
| Tuvalu* | 0.10 |
| United States Virgin Islands | 0.06 |
| United Arab Emirates | 0.71 |
| Vanuatu | 0.001 |
| Venezuela | 0.28 |
| Vietnam* | 0.16 |
| Wallis and Futuna | 0.01 |
| Yemen | 0.41 |
| ^*^ The reef to total landed value % for these countries were based on regional averages. | |

| Table S3. Estimated number of reef fishers for reef countries and territories worldwide in 2010. Estimates reported for countries marked ‘*’ are from alternate sources. | | | |
| --- | --- | --- | --- |
| **Country** | **Region** | **No. of reef fishers** | **Year and source of alternate estimate** |
| American Samoa | Western Pacific | 1,847 |  |
| Anguilla | Eastern Pacific/Atlantic | 208 |  |
| Antigua and Barbuda* | Eastern Pacific/Atlantic | 864 | 2005; [1] |
| Aruba | Eastern Pacific/Atlantic | 1,018 |  |
| Australia | Western Pacific | 29,593 |  |
| Bahamas* | Eastern Pacific/Atlantic | 12,000 | 2001; [2] |
| Bahrain* | Middle East/NE Africa | 7,200 | 2001; [1] |
| Bangladesh | Indian Ocean | 230,498 |  |
| Barbados | Eastern Pacific/Atlantic | 566 |  |
| Belize | Eastern Pacific/Atlantic | 6,926 |  |
| Bermuda | Eastern Pacific/Atlantic | 2,158 |  |
| Brazil | Eastern Pacific/Atlantic | 144,433 |  |
| British Virgin Islands | Eastern Pacific/Atlantic | 1,579 |  |
| Brunei Darsm | Southeast Asia | 920 |  |
| Cambodia | Southeast Asia | 14,364 |  |
| Cayman Islands | Eastern Pacific/Atlantic | 1,318 |  |
| China^1^ | Southeast Asia | 189,467 |  |
| Colombia | Eastern Pacific/Atlantic | 12,188 |  |
| Comoros | Indian Ocean | 12,077 |  |
| Cook Islands | Western Pacific | 3,971 |  |
| Costa Rica | Eastern Pacific/Atlantic | 12,303 |  |
| Cuba* | Eastern Pacific/Atlantic | 11,890 | 2002; [1] |
| Djibouti | Middle East/NE Africa | 901 |  |
| Dominica | Eastern Pacific/Atlantic | 1,377 |  |
| Dominican Republic* | Eastern Pacific/Atlantic | 9,000 | 2010; [3] |
| Ecuador | Eastern Pacific/Atlantic | 10,439 |  |
| Egypt | Middle East/NE Africa | 205,260 |  |
| Eritrea | Middle East/NE Africa | 11,255 |  |
| Federated States of Micronesia | Western Pacific | 35,960 |  |
| Fiji* | Western Pacific | 43,475 | 2007; [4] |
| Florida & US Gulf of Mexico | Eastern Pacific/Atlantic | 45,000 |  |
| French Polynesia | Western Pacific | 21,495 |  |
| Grenada | Eastern Pacific/Atlantic | 1,953 |  |
| Guadeloupe | Eastern Pacific/Atlantic | 2,446 |  |
| Guam | Western Pacific | 1,817 |  |
| Haiti | Eastern Pacific/Atlantic | 55,045 |  |
| Hawaii | Western Pacific | 42,480 |  |
| Honduras | Eastern Pacific/Atlantic | 12,454 |  |
| India | Indian Ocean | 958,530 |  |
| Indonesia | Southeast Asia | 1,657,757 |  |
| Iran | Middle East/NE Africa | 15,953 |  |
| Israel | Middle East/NE Africa | 400 |  |
| Jamaica | Eastern Pacific/Atlantic | 20,000 | 2002; [1] |
| Japan^1^ | Southeast Asia | 30,576 |  |
| Jordan | Middle East/NE Africa | 90 |  |
| Kenya | Indian Ocean | 12,938 |  |
| Kiribati | Western Pacific | 14,260 |  |
| Kuwait | Middle East/NE Africa | 3,566 |  |
| Madagascar | Indian Ocean | 61,906 |  |
| Malaysia | Southeast Asia | 83,720 |  |
| Maldives | Indian Ocean | 30,223 |  |
| Marshall Islands | Western Pacific | 21,743  21,743 |  |
| Martinique* | Eastern Pacific/Atlantic | 2,500 | 2005; [5] |
| Mauritius | Indian Ocean | 7,127 |  |
| Mayotte | Indian Ocean | 1,005 |  |
| Mexico | Eastern Pacific/Atlantic | 64,705 |  |
| Mozambique | Indian Ocean | 50,326 |  |
| Myanmar | Southeast Asia | 123,746 |  |
| Nauru | Western Pacific | 292 |  |
| Netherland Antilles | Eastern Pacific/Atlantic | 790 |  |
| New Caledonia | Western Pacific | 23,539 |  |
| Nicaragua | Eastern Pacific/Atlantic | 6,755 |  |
| Niue | Western Pacific | 607 |  |
| Northern Mariana Islands | Western Pacific | 603 |  |
| Oman | Middle East/NE Africa | 10,287 |  |
| Palau | Western Pacific | 3,795 |  |
| Panama | Eastern Pacific/Atlantic | 6,551 |  |
| Papua New Guinea | Western Pacific | 107,952 |  |
| Philippines | Southeast Asia | 911,754 |  |
| Puerto Rico* | Eastern Pacific/Atlantic | 1,163 | 2002; [6] |
| Qatar | Middle East/NE Africa | 4,505 |  |
| Reunion | Indian Ocean | 1,060 |  |
| St. Kitts and Nevis* | Eastern Pacific/Atlantic | 488 | Undated; [7] |
| St. Lucia | Eastern Pacific/Atlantic | 1,040 |  |
| St. Vincent and the Grenadines | Eastern Pacific/Atlantic | 587 |  |
| Samoa | Western Pacific | 3,586 |  |
| Saudi Arabia | Middle East/NE Africa | 24,500 24,500 |  |
| Seychelles* | Indian Ocean | 2,000 | 2005; [1] |
| Solomon Islands | Western Pacific | 58,930 |  |
| Somalia | Indian Ocean | 3,694 |  |
| Sri Lanka | Indian Ocean | 22,417 22,417 |  |
| Sudan | Middle East/NE Africa | 27,254 27,254 |  |
| Taiwan^1^ | Southeast Asia | 26,516 |  |
| Tanzania | Indian Ocean | 108,789 |  |
| Thailand | Southeast Asia | 99,807 |  |
| Timor-Leste* | Southeast Asia | 5,415 | 2005; [8] |
| Tokelau | Western Pacific | 179 |  |
| Tonga | Western Pacific | 7,170 |  |
| Trinidad and Tobago | Eastern Pacific/Atlantic | 6,005 |  |
| Turks and Caicos Islands | Eastern Pacific/Atlantic | 2,524 |  |
| Tuvalu | Western Pacific | 2,708 |  |
| United States Virgin Islands | Eastern Pacific/Atlantic | 981 |  |
| United Arab Emirates | Middle East/NE Africa | 12,385 |  |
| Vanuatu | Western Pacific | 9,410 |  |
| Venezuela | Eastern Pacific/Atlantic | 21,291 |  |
| Vietnam | Southeast Asia | 204,546 |  |
| Wallis and Futuna | Western Pacific | 10,357 10,357 |  |
| Yemen | Middle East/NE Africa | 20,993 |  |
| **Total** |  | **6,145,200** |  |
| ^1^ The north Asian countries of China, Japan, and Taiwan are catergorised under Southeast Asia as per the grouping by [5]. | | | |

| Table S4. Comparison of fisher estimates from current study and alternate independent sources. | | | |
| --- | --- | --- | --- |
| **Country/Territory** | **Estimate from this study** | **Estimate from alternate source** | **Source** |
| Antigua Barbuda | *4,315 | 864 | [1] |
| Bahamas | *40,051 | 12,000 | [2] |
| Bahrain | *36,969 | 7,200 | [2] |
| Cuba | *333,826 | 11,890 | [1] |
| Dominican Republic | *121,869 | 9,000 | [3] |
| Fiji | *280,059 | 43,475 | [4] |
| French Polynesia | 21,495 | 3,500 | [5] |
| Jamaica | 43,697 | 20,000 | [1] |
| Madagascar | 61,906 | 50,000 | [9] |
| Martinique | *2,148 | 2,500 | [6] |
| Mayotte | 1,005 | 3,600 | [10] |
| Mozambique | 50,326 | 90,000 | [11] |
| Nauru | 292 | 4,451 | [12] |
| Niue | 607 | 597 | [13] |
| Palau | 3,795 | 1,100 | [14] |
| Philippines | 911,754 | 1,000,000 | [15] |
| Puerto Rico | *10,320 | 1,163 | [7] |
| Samoa | 3,586 | 11,700 | [16] |
| Seychelles | *15,355 | 2,000 | [1] |
| St. Kitts and Nevis | *1,487 | 488 | [17] |
| Tanzania | 108,789 | 12,500 | [11] |
| Timor Leste | *1,290 | 5,415 | [8] |
| Tonga | 7,170 | 2,007 | [18] |
| * These estimates exceed 70% of rural coastal population and were replaced with estimates from alternate sources for the final computation of total reef fisher numbers. See text and Table S3. | | | |

**References**

[1] FAO (2011) Fishery and Aquaculture Country Profiles. Available: http://www.fao.org/fishery/countryprofiles/search/en. Accessed 18 Februrary 2011.

[2] CARICOM (2001) Report of the multidisciplinary survey of the fisheries of the Bahamas. Commission of the European Union Lome IV-Project No. 7: ACP:RPR:385. Belize City, Belize: CARICOM Fisheries Unit. 99 p.

[3] Wielgus J, Cooper E, Torres R, Burke L (2010) Coastal Capital: Dominican Republic. Case studies on the economic value of coastal ecosystems in the Dominican Republic. Working Paper. Washington DC: World Resources Institute. 42 p.

[4] WWF (2012) Problems in the Coral Triangle. Available: http://wwf.panda.org/what_we_do/where_we_work/coraltriangle/problems/. Accessed 15 August 2012.

[5] Gabrié C, You H (2006) L’état de l'environnement en Polynésie française. Papeete: Ministère de l'environnement de la Polynésie française.

[6] Wilkinson C, Souter D (2008) Status of Caribbean coral reefs after bleaching and hurricanes in 2005. Townsville: Global Coral Reef Monitoring Network and Reef and Rainforest Research Centre. 152 p.

[7] Garcia-Sais J, Appeldoorn R, Battista T, Bauer L, Bruckner A, et al. (2008) The state of coral reef ecosystems of Puerto Rico. In: Waddell J, Clarke A, editors. The state of coral reef ecosystems of the United States and Pacific freely associated states: 2008. NOAA Technical Memorandum NOS NCCOS 73. Silver Spring, MD: NOAA/NCCOS Center for coastal monitoring and assessment's biogeography team. pp. 75-116.

[8] Munday P (2000) The status of coral reefs in Papua New Guinea. Townsville: Australian Institute of Marine Science. 91 p.

[9] Gabrie C, Vasseur P, Randriamiarana H, Maharavo J, Mara E (2000) The coral reefs of Madagascar. In: Obura DO, editor. Coral Reefs of the Indian Ocean: Their Ecology and Conservation. Oxford: Oxford University Press. pp. 411–444.

[10] Spalding M, Ravilious C, Green E (2001) World atlas of coral reefs Berkeley, USA: University of California Press.

[11] Whittingham E, Campbell J, Townsley P (2003) Poverty and Reefs. Paris: DFID–IMM–IOC/UNESCO. 260 p.

[12] Sauni S, Vunisea A, Kronen M, Friedman K, Magron F, et al. (2007) Nauru country report: Profile and results from in-country survey work (October and November 2005). Noumea: Pacific Regional Oceanic and Coastal Fisheries Development Programme (PROCFish/C/CoFish) / Secretariat of the Pacific Community. 137 p.

[13] Kronen M, Fisk D, Pinca S, Magron F, Friedman K, et al. (2009) Niue country report: Profile and results from in-country survey work (May to June 2005). Noumea: Pacific Regional Oceanic and Coastal Fisheries Development Programme (PROCFish/C/CoFish) / Secretariat of the Pacific Community.

173 p.

[14] Friedman K, Kronen M, Pinca S, Lasi F, Pakoa K, et al. (2009) Palau country report: Profiles and results from survey work at Ngarchelong, Ngatpang, Airai and Koror (April to June 2007). Noumea: Pacific Regional Oceanic and Coastal Fisheries Development Programme (PROCFish/C/CoFish) / Secretariat of the Pacific Community. 411 p.

[15] White AT, Cruz-Trinidad A (1998) The Values of Philippines Coastal Resources: Why Protection and Management are Critical. Cebu City: Coastal Resource Management Project, Cebu City. 96 p.

[16] Vunisea A, Friedman K, Awira R, Kronen M, Pinca S, et al. (2008) Samoa country report: profiles and results from survey work at Manono-Uta, Salelavalu, Vailoa and Vaisala (June 2005 and August/September 2005). Noumea: Pacific Regional Oceanic and Coastal Fisheries Development Programme (PROCFish/C/CoFish) / Secretariat of the Pacific Community. 349 p.

[17] ACP Fish II (2012) Africa Caribbean Pacific Fish II - St. Kitts and Nevis. Available: http://acpfish2-eu.org/index.php?page=st-kitts-and-nevis&hl=en. Accessed 20 August 2012.

[18] Friedman K, Pinca S, Kronen M, Boblin P, Chapman L, et al. (2009) Tonga country report: profiles and results from survey work at Ha'atafu, Manuka, Koulo and Lofanga (November and December 2001; March to June 2002; April to June, September and October 2008). Noumea: Pacific Regional Oceanic and Coastal Fisheries Development Programme (PROCFish/C/CoFish) / Secretariat of the Pacific Community. 369 p.
